# Prevalence of Self-Reported Hypertension and Antihypertensive Medication Use Among Adults Aged ≥18 Years — United States, 2011–2015

**DOI:** 10.15585/mmwr.mm6707a4

**Published:** 2018-02-23

**Authors:** Jing Fang, Cathleen Gillespie, Carma Ayala, Fleetwood Loustalot

**Affiliations:** 1 Division for Heart Disease and Stroke Prevention, National Center for Chronic Disease Prevention and Health Promotion, CDC.

Hypertension, which affects nearly one third of adults in the United States, is a major risk factor for heart disease and stroke (*1*), and only approximately half of those with hypertension have their hypertension under control (*2*). The prevalence of hypertension is highest among non-Hispanic blacks, whereas the prevalence of antihypertensive medication use is lowest among Hispanics (*1*). Geographic variations have also been identified: a recent report indicated that the Southern region of the United States had the highest prevalence of hypertension as well as the highest prevalence of medication use (*3*). Using data from the Behavioral Risk Factor Surveillance System (BRFSS), this study found minimal change in state-level prevalence of hypertension awareness and treatment among U.S. adults during the first half of the current decade. From 2011 to 2015, the age-standardized prevalence of self-reported hypertension decreased slightly, from 30.1% to 29.8% (p = 0.031); among those with hypertension, the age-standardized prevalence of medication use also decreased slightly, from 63.0% to 61.8% (p<0.001). Persistent differences were observed by age, sex, race/ethnicity, level of education, and state of residence. Increasing hypertension awareness, as well as increasing hypertension control through lifestyle changes and consistent antihypertensive medication use, requires diverse clinical and public health intervention.

BRFSS is a state-based telephone survey of noninstitutionalized adults aged ≥18 years.* Data for this study were taken from the fixed core questions asked every year and the rotating core questions asked every other year. Hypertension awareness questions, included in the rotating core, were asked in odd years. New survey methods were introduced to the BRFSS in 2011; thus, available data from 2011 to 2015 were used for trend analyses. The median state-specific response rates in 2011, 2013, and 2015 were 49.7% (range = 33.8%–64.1%), 45.9% (29.0%–59.2%), and 47.2% (33.9%–61.1%), respectively.^†^

Self-reported diagnosed hypertension was ascertained by an affirmative response to the question “Have you ever been told by a doctor, nurse, or other health professional that you have high blood pressure?” To determine whether persons with hypertension were being treated, respondents who answered “yes” were asked “Are you currently taking medicine for your high blood pressure?” Hypertension and treatment were assessed by age group (18–44 years, 45–64 years, and ≥65 years), sex, race/ethnicity (non-Hispanic whites [whites]; non-Hispanic blacks [blacks]; Hispanics; non-Hispanic Asians [Asians]; Native Hawaiian/Pacific Islanders [NH/PIs]; non-Hispanic American Indian/Alaskan Natives [AI/ANs]; and non-Hispanic others [others]), highest level of education attained (less than high school graduate, high school graduate, some college, college graduate or higher), and state of residence. Estimates were directly age-standardized to the 2000 U.S. standard population. Changes over time were assessed using t-tests for the differences from 2011 to 2015. Because of a large difference in the age distribution between persons with hypertension and the general population, both age-standardized and crude estimates were calculated. All analyses were conducted using statistical software to account for the complex sampling design.

Overall, 497,967, 483,865, and 434,382 participants were interviewed in 2011, 2013, and 2015, respectively. After excluding participants who were pregnant (0.5%–0.6%), missing data for hypertension variables (0.3%–0.4%) and other covariates (2.0%–2.8%), the final analytic samples for 2011, 2013 and 2015 were 483,120 (97% of original sample), 465,739 (96%), and 418,317 (96%), respectively. From 2011 to 2015, the overall age-standardized prevalence of self-reported hypertension decreased from 30.1% to 29.8% (p = 0.031). Hypertension prevalence was higher in 2015 among adults aged ≥65 years (61.7%), men (32.5%), blacks (40.3%), and persons with less than high school education (35.1%) compared with younger adults, women (27.1%), Asians (24.6%), and persons with higher levels of education (Table 1). Statistically significant, but minimal, declines in the prevalence of hypertension from 2011 to 2015 were observed among women (28.1% to 27.1%), persons aged ≥65 years (62.2% to 61.7%), and persons with some college education (30.5% to 29.8%). In contrast, an increase in hypertension prevalence was observed among persons with less than high school education (34.1% to 35.1%).

**TABLE 1:** Age-standardized prevalence of self-reported hypertension among adults aged ≥18 years by sociodemographic characteristics and state* — Behavioral Risk Factor Surveillance System, United States 2011–2015

Characteristic	% (95% CI)			Change 2011–2015	
	2011	2013	2015	%	p-value^†^
**Total**	**30.1 (29.9–30.4)**	**30.6 (30.3–30.8)**	**29.8 (29.5–30.0)**	**-0.3**	**0.031**
**Sex**					
Male	32.1 (31.7–32.4)	32.8 (32.4–33.2)	32.5 (32.1–32.9)	0.4	0.096
Female	28.1 (27.8–28.4)	28.3 (28.0–28.6)	27.1 (26.8–27.4)	-1.0	<0.001
**Age group (yrs)**					
18–44	14.1 (13.8–14.5)	14.2 (13.8–14.5)	13.7 (13.4–14.1)	-0.4	0.210
45–64	40.2 (39.8–40.6)	41.1 (40.7–41.6)	40.2 (39.7–40.6)	-0.1	0.496
≥65	62.2 (61.7–62.7)	63.0 (62.5–63.5)	61.7 (61.1–62.2)	-0.5	0.039
**Race/Ethnicity**					
White, non-Hispanic	29.0 (28.7–29.3)	29.3 (29.0–29.5)	28.8 (28.5–29.0)	-0.2	0.102
Black, non-Hispanic	41.2 (40.4–42.0)	41.4 (40.6–42.2)	40.3 (39.5–41.1)	-0.9	0.094
Asian, non-Hispanic	25.4 (23.9–27.0)	27.0 (25.2–28.8)	24.6 (22.9–26.3)	-0.9	0.707
Native Hawaiian/Pacific Islander	34.6 (29.6–39.9)	28.8 (24.5–33.6)	32.8 (28.6–37.3)		0.523
American Indian/Alaska Native	36.2 (34.0–38.4)	34.2 (32.2–36.3)	35.0 (33.1–37.1)	-1.1	0.540
Hispanic	28.3 (27.5–29.2)	29.7 (28.7–30.6)	28.0 (27.1–28.9)	-0.3	0.789
Other^§^	27.7 (25.5–30.0)	29.2 (26.7–31.7)	28.0 (25.5–30.8)	0.3	0.562
**Education**					
Less than high school	34.1 (33.3–34.9)	36.2 (35.3–37.1)	35.1 (34.2–36.0)	1.0	0.019
High school graduate	32.2 (31.7–32.6)	32.0 (31.6–32.4)	31.9 (31.4–32.3)	-0.3	0.574
Some college	30.5 (30.1–30.9)	31.0 (30.5–31.4)	29.8 (29.3–30.2)	-0.7	0.012
College graduate or higher	25.2 (24.8–25.5)	25.4 (25.0–25.8)	24.9 (24.5–25.3)	-0.3	0.136
**State**					
Alabama	37.9 (36.5–39.4)	37.6 (36.0–39.3)	37.6 (36.2–39.0)	-0.4	0.663
Alaska	30.8 (28.8–32.9)	30.2 (28.5–32.0)	27.9 (26.0–29.9)	-2.9	0.030
Arizona	26.3 (24.6–28.2)	29.5 (27.2–32.0)	28.5 (27.2–29.9)	2.2	0.053
Arkansas	33.7 (31.9–35.6)	36.4 (34.5–38.3)	36.7 (34.5–39.0)	3.1	0.033
California	27.8 (27.0–28.6)	28.2 (27.2–29.3)	27.7 (26.7–28.6)	-0.2	0.703
Colorado	24.8 (23.9–25.7)	25.8 (25.0–26.7)	24.6 (23.6–25.7)	-0.1	0.688
Connecticut	27.6 (26.3–29.0)	28.3 (27.0–29.6)	27.0 (26.0–28.1)	-0.6	0.518
Delaware	32.5 (30.8–34.3)	32.6 (31.0–34.2)	31.2 (29.4–33.2)	-1.3	0.262
District of Columbia	31.0 (29.3–32.8)	30.2 (28.6–32.0)	31.0 (28.7–33.4)	0.0	0.927
Florida	30.6 (29.4–31.8)	30.6 (29.6–31.7)	29.4 (28.1–30.7)	-1.3	0.081
Georgia	32.4 (31.2–33.7)	34.5 (33.2–35.8)	35.0 (33.4–36.6)	2.6	0.020
Hawaii	26.8 (25.5–28.2)	26.2 (24.9–27.5)	29.7 (28.3–31.2)	2.9	0.013
Idaho	28.9 (27.3–30.5)	27.7 (26.2–29.2)	29.6 (28.0–31.3)	0.8	0.528
Illinois	30.1 (28.5–31.7)	28.7 (27.2–30.3)	28.9 (27.6–30.2)	-1.1	0.333
Indiana	31.3 (30.1–32.5)	31.6 (30.5–32.7)	30.0 (28.5–31.6)	-1.3	0.228
Iowa	27.5 (26.3–28.6)	28.6 (27.4–29.8)	27.8 (26.5–29.1)	0.3	0.798
Kansas	29.4 (28.7–30.2)	29.4 (28.8–30.1)	29.6 (29.0–30.3)	0.2	0.742
Kentucky	36.1 (34.7–37.5)	36.6 (35.4–37.9)	36.3 (34.8–37.9)	0.2	0.726
Louisiana	37.3 (36.0–38.6)	38.0 (36.1–39.9)	37.5 (35.8–39.1)	0.2	0.881
Maine	28.6 (27.6–29.5)	29.2 (27.9–30.4)	29.0 (27.7–30.2)	0.4	0.431
Maryland	29.9 (28.7–31.2)	30.9 (29.8–32.0)	30.6 (29.1–32.1)	0.7	0.356
Massachusetts	27.6 (26.7–28.5)	27.1 (26.1–28.1)	27.2 (26.0–28.3)	-0.4	0.695
Michigan	32.1 (30.9–33.3)	31.8 (30.7–32.8)	30.0 (29.0–31.1)	-2.0	0.008
Minnesota	25.2 (24.3–26.1)	25.4 (24.2–26.6)	24.2 (23.5–25.0)	-1.0	0.115
Mississippi	37.8 (36.6–39.1)	38.3 (36.8–39.8)	40.1 (38.4–41.8)	2.2	0.063
Missouri	32.3 (30.8–33.8)	29.5 (27.9–31.1)	31.5 (30.1–33.0)	-0.7	0.356
Montana	27.5 (26.3–28.8)	26.3 (25.2–27.4)	25.9 (24.4–27.4)	-1.6	0.056
Nebraska	26.9 (26.3–27.6)	28.4 (27.5–29.5)	27.7 (26.8–28.7)	0.8	0.227
Nevada	30.6 (28.6–32.7)	29.4 (27.5–31.5)	26.7 (24.5–29.0)	-3.9	0.009
New Hampshire	28.7 (27.3–30.2)	27.1 (25.8–28.5)	25.8 (24.4–27.2)	-2.9	0.005
New Jersey	28.8 (27.8–29.8)	28.5 (27.5–29.5)	28.2 (27.1–29.4)	-0.6	0.551
New Mexico	27.0 (26.0–28.1)	27.4 (26.3–28.6)	28.0 (26.6–29.4)	0.9	0.341
New York	29.1 (27.9–30.5)	29.4 (28.2–30.6)	27.2 (26.2–28.2)	-1.9	0.018
North Carolina	30.9 (29.8–32.1)	33.4 (32.2–34.6)	32.8 (31.6–34.0)	1.9	0.028
North Dakota	27.4 (26.1–28.8)	27.6 (26.4–28.8)	28.9 (27.5–30.3)	1.5	0.100
Ohio	30.4 (29.2–31.6)	30.5 (29.4–31.6)	31.2 (29.9–32.5)	0.8	0.417
Oklahoma	33.9 (32.7–35.2)	35.6 (34.4–36.9)	33.9 (32.5–35.3)	-0.1	0.895
Oregon	27.8 (26.5–29.2)	29.5 (28.0–31.1)	27.5 (26.1–28.9)	-0.3	0.721
Pennsylvania	28.6 (27.5–29.8)	30.4 (29.3–31.5)	29.0 (27.6–30.5)	0.3	0.602
Rhode Island	30.6 (29.2–32.0)	31.0 (29.6–32.4)	29.2 (27.7–30.6)	-1.4	0.230
South Carolina	34.1 (32.9–35.3)	35.5 (34.3–36.7)	34.7 (33.6–35.9)	0.6	0.489
South Dakota	28.7 (27.0–30.4)	27.9 (26.4–29.4)	27.5 (25.9–29.1)	-1.2	0.244
Tennessee	37.0 (34.6–39.4)	36.2 (34.6–37.9)	35.3 (33.6–36.9)	-1.7	0.360
Texas	31.7 (30.5–32.9)	31.3 (30.1–32.6)	29.2 (28.0–30.4)	-2.5	0.004
Utah	25.0 (24.2–25.9)	25.7 (24.8–26.5)	25.0 (24.1–25.8)	-0.1	0.891
Vermont	26.7 (25.5–28.0)	27.7 (26.4–29.0)	25.7 (24.5–27.0)	-1.0	0.299
Virginia	30.0 (28.6–31.5)	30.9 (29.7–32.2)	31.5 (30.3–32.8)	1.5	0.193
Washington	29.4 (28.3–30.5)	28.9 (27.9–30.0)	28.1 (27.2–29.0)	-1.3	0.036
West Virginia	33.8 (32.4–35.3)	36.8 (35.4–38.3)	38.6 (37.1–40.0)	4.8	<0.001
Wisconsin	27.0 (25.3–28.7)	29.7 (28.1–31.4)	26.8 (25.4–28.3)	-0.2	0.918
Wyoming	27.6 (26.2–29.0)	27.0 (25.6–28.4)	27.7 (26.0–29.5)	0.2	0.887

**Abbreviation:** CI = confidence interval.

* Directly standardized to the 2000 U.S. standard population.

^†^ Adjusted for sex, age group, and race/ethnicity.

^§^ Includes participants of multiple racial/ethnic groups.

By state, the age-standardized prevalence of self-reported hypertension ranged from 24.2% in Minnesota to 40.1% in Mississippi in 2015 (Table 1). From 2011 to 2015, significant increases in the prevalence of hypertension were observed in five states (Arkansas, Georgia, Hawaii, North Carolina, and West Virginia) and significant decreases were observed in six states (Michigan, Nevada, New Hampshire, New York, Texas, and Washington). In 2015, hypertension prevalence was, in general, higher in the Southern states and lower in the Western states (Figure).

**FIGURE:**
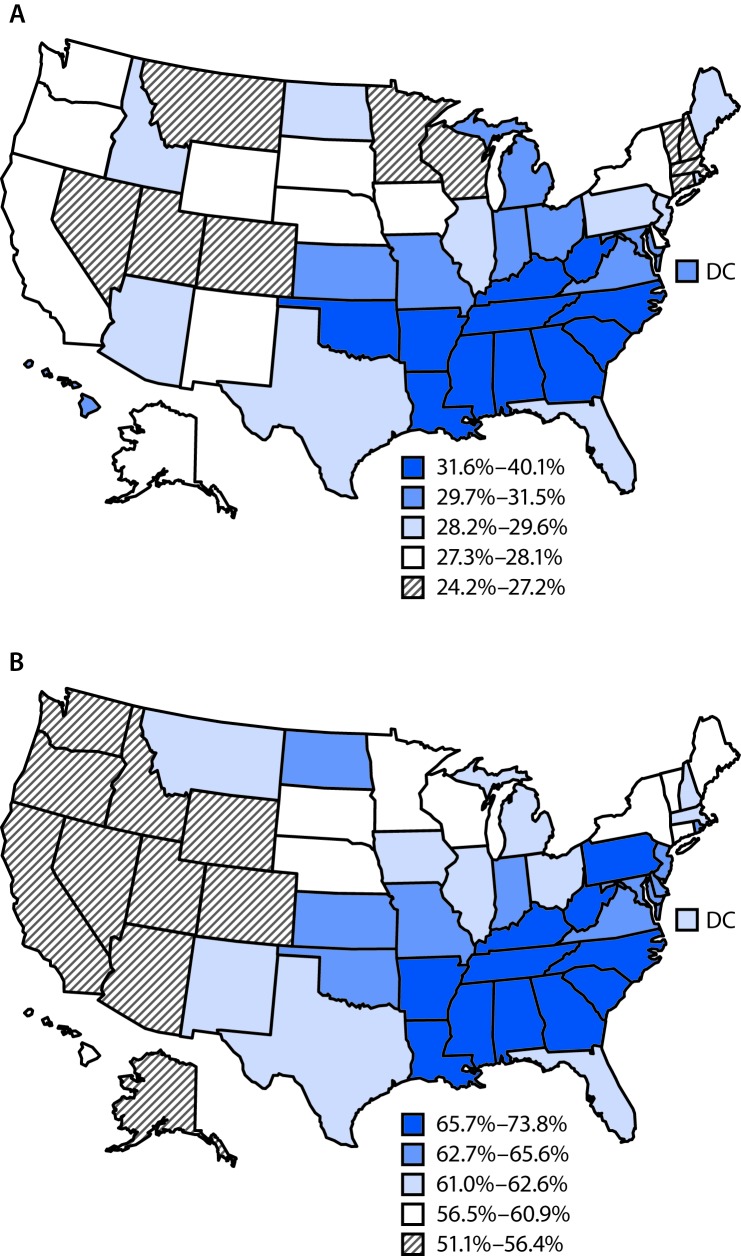
Age-standardized prevalence of self-reported hypertension among adults (A) and use of antihypertensive medication among adults with self-reported hypertension (B), by state — Behavioral Risk Factor Surveillance System, 50 states and the District of Columbia (DC), 2015

Among participants with self-reported hypertension, the age-standardized prevalences of antihypertensive medication use in 2011, 2013, and 2015 were 63.0%, 62.0%, and 61.8%, respectively (p<0.001, Table 2). In 2015, the prevalence of medication use was higher among women (66.8%), adults aged ≥65 years (93.1%), and blacks (60.7%), and lower among men (58.5%), adults aged 18–44 years (41.2%), and Hispanics (55.4%). From 2011 to 2015, significant decreases in antihypertensive medication use among persons with self-reported hypertension were observed among both men and women, persons aged ≥65 years, whites, and high school graduates, as well as those with any college education. By state, a significant decrease in the prevalence of medication use was observed in Connecticut, Hawaii, North Carolina, South Carolina, Texas, Utah, and West Virginia. In 2015, the prevalence of medication use among persons with self-reported hypertension was highest in Louisiana (73.8%) and lowest in Idaho (51.1%). In general, the prevalence of medication use was higher in the Southern states and lower in the Western states (Figure).

**TABLE 2:** Age-standardized prevalence of use of antihypertensive medication among adults aged ≥18 years with self-reported hypertension, by sociodemographic characteristics and state* — Behavioral Risk Factor Surveillance System, United States, 2011–2015

Characteristic	% (95% CI)			Change 2011–2015	
	2011	2013	2015	%	p-value^†^
**Total**	**63.0 (62.3–63.8)**	**62.0 (61.3–62.7)**	**61.8 (61.0–62.5)**	**-1.3**	**<0.001**
**Sex**					
Male	59.6 (58.7–60.6)	58.3 (57.4–59.2)	58.5 (57.6–59.4)	-1.1	0.029
Female	68.2 (67.1–69.2)	67.1 (66.0–68.2)	66.8 (65.7–67.9)	-1.3	0.007
**Age group (yrs)**					
18–44	42.9 (41.6–44.2)	41.4 (40.1–42.6)	41.2 (39.9–42.5)	-1.7	0.180
45–64	81.2 (80.6–81.8)	80.7 (80.1–81.3)	80.3 (79.7–80.9)	-0.9	0.048
≥65	93.9 (93.6–94.2)	93.1 (92.8–93.4)	93.1 (92.8–93.4)	-0.8	<0.001
**Race/Ethnicity**					
White, non-Hispanic	63.1 (62.3–64.0)	61.9 (61.1–62.7)	60.7 (59.8–61.5)	-2.5	<0.001
Black, non-Hispanic	69.7 (67.8–71.4)	68.7 (67.0–70.4)	70.7 (68.8–72.4)	1.0	0.146
Asian, non-Hispanic	59.9 (55.6–64.0)	58.2 (53.3–62.8)	62.7 (58.6–66.6)	2.8	0.491
Native Hawaiian/Pacific Islander	63.8 (53.4–73.0)	56.3 (47.3–65.0)	55.1 (46.4–63.5)	-8.7	0.148
American Indian/Alaska Native	61.8 (57.5–66.0)	62.0 (57.8–66.0)	61.2 (56.5–65.6)	-0.7	0.867
Hispanic	54.6 (52.5–56.8)	55.4 (53.3–57.5)	55.4 (53.3–57.5)	0.8	0.952
Other^§^	61.2 (54.2–67.7)	57.4 (50.6–64.0)	60.6 (53.1–67.7)	-0.5	0.771
**Education**					
Less than high school	60.0 (58.0–62.0)	59.8 (57.7–61.8)	60.3 (58.0–62.5)	0.3	0.845
High school graduate	64.3 (63.0–65.6)	62.7 (61.4–63.9)	61.8 (60.5–63.1)	-2.4	0.031
Some college	62.6 (61.4–63.9)	61.5 (60.2–62.7)	61.8 (60.5–63.0)	-0.8	0.128
College graduate or higher	64.1 (62.8–65.4)	63.4 (62.2–64.6)	62.5 (61.2–63.7)	-1.6	0.002
**State**					
Alabama	72.7 (69.3–75.9)	71.2 (67.0–75.0)	70.4 (66.9–73.6)	-2.4	0.392
Alaska	52.3 (47.8–56.7)	48.6 (44.5–52.7)	51.7 (46.3–57.1)	-0.5	0.455
Arizona	56.3 (50.6–61.8)	50.8 (45.6–55.9)	56.1 (52.2–60.0)	-0.1	0.701
Arkansas	67.6 (62.0–72.9)	66.5 (61.9–70.8)	66.5 (60.7–71.8)	-1.2	0.958
California	53.8 (51.5–56.2)	54.0 (51.1–56.8)	53.3 (50.8–55.8)	-0.5	0.286
Colorado	51.6 (48.6–54.5)	54.6 (51.9–57.4)	54.0 (50.6–57.4)	2.4	0.916
Connecticut	63.2 (58.9–67.3)	58.3 (54.1–62.3)	58.1 (54.2–61.9)	-5.1	0.001
Delaware	63.9 (59.2–68.3)	70.1 (65.2–74.6)	63.5 (57.6–69.0)	-0.4	0.717
District of Columbia	64.2 (58.5–69.5)	59.7 (54.7–64.4)	61.0 (54.4–67.2)	-3.2	0.588
Florida	61.9 (58.3–65.3)	60.8 (57.8–63.8)	62.6 (58.6–66.3)	0.7	0.665
Georgia	68.5 (64.8–72.0)	66.8 (63.5–70.0)	65.7 (61.3–69.9)	-2.8	0.890
Hawaii	65.6 (60.9–70.1)	62.8 (58.3–67.2)	58.9 (54.9–62.7)	-6.7	0.002
Idaho	52.6 (48.6–56.6)	54.6 (49.7–59.5)	51.1 (46.9–55.2)	-1.5	0.159
Illinois	62.5 (57.6–67.2)	60.7 (55.8–65.4)	62.0 (57.6–66.2)	-0.6	0.690
Indiana	65.4 (61.8–68.8)	64.9 (61.6–68.0)	63.1 (58.1–67.8)	-2.3	0.339
Iowa	58.7 (55.2–62.1)	61.2 (57.3–65.0)	61.4 (57.0–65.7)	2.7	0.395
Kansas	62.1 (59.9–64.2)	62.7 (60.6–64.7)	62.6 (60.6–64.6)	0.5	0.655
Kentucky	67.6 (64.2–70.8)	69.2 (66.1–72.2)	68.2 (64.2–71.9)	0.6	0.471
Louisiana	73.9 (70.7–76.9)	70.4 (65.8–74.7)	73.8 (69.7–77.6)	-0.1	0.828
Maine	61.1 (57.9–64.2)	64.7 (60.7–68.5)	57.1 (53.2–60.9)	-4.0	0.094
Maryland	68.8 (64.9–72.4)	66.0 (62.6–69.3)	63.4 (58.7–67.8)	-5.4	0.054
Massachusetts	61.1 (58.2–63.9)	56.5 (53.3–59.6)	62.1 (58.4–65.6)	0.9	0.784
Michigan	62.1 (58.8–65.3)	58.0 (55.0–60.9)	61.5 (58.3–64.5)	-0.7	0.908
Minnesota	60.9 (57.6–64.0)	59.9 (56.2–63.6)	60.7 (57.8–63.4)	-0.2	0.851
Mississippi	71.9 (68.9–74.7)	73.7 (70.1–77.1)	72.1 (68.0–75.9)	0.2	0.838
Missouri	64.8 (60.6–68.8)	72.7 (67.3–77.5)	65.6 (61.3–69.7)	0.8	0.607
Montana	55.2 (51.4–58.9)	56.3 (52.9–59.5)	61.8 (56.2–67.0)	6.6	0.118
Nebraska	60.7 (58.3–62.9)	64.0 (60.6–67.2)	60.2 (56.9–63.4)	-0.5	0.685
Nevada	54.8 (49.4–60.0)	59.9 (53.9–65.6)	52.1 (45.8–58.4)	-2.7	0.363
New Hampshire	56.6 (52.7–60.5)	56.7 (52.6–60.6)	60.2 (54.7–65.5)	3.6	0.398
New Jersey	60.1 (57.1–63.0)	59.3 (56.3–62.2)	64.0 (60.0–67.7)	3.9	0.506
New Mexico	60.9 (57.1–64.6)	57.3 (53.7–60.7)	61.6 (56.7–66.2)	0.7	0.315
New York	61.6 (57.4–65.7)	59.8 (56.2–63.3)	60.9 (57.3–64.3)	-0.7	0.080
North Carolina	74.0 (70.3–77.3)	63.1 (59.9–66.2)	68.2 (64.5–71.6)	-5.8	0.007
North Dakota	61.4 (56.9–65.8)	64.1 (59.9–68.1)	65.2 (60.3–69.8)	3.8	0.069
Ohio	65.9 (62.2–69.4)	64.5 (61.2–67.6)	62.4 (58.7–66.0)	-3.5	0.686
Oklahoma	68.6 (65.2–71.7)	68.9 (65.7–71.8)	64.8 (60.7–68.8)	-3.7	0.054
Oregon	54.9 (51.0–58.7)	56.1 (51.5–60.6)	54.1 (49.8–58.3)	-0.8	0.545
Pennsylvania	62.9 (59.4–66.2)	64.2 (61.2–67.2)	65.8 (61.2–70.2)	3.0	0.164
Rhode Island	62.1 (57.9–66.2)	64.4 (60.2–68.4)	63.3 (57.9–68.3)	1.2	0.642
South Carolina	72.3 (69.1–75.3)	68.8 (65.7–71.8)	67.5 (64.3–70.6)	-4.8	0.020
South Dakota	60.2 (54.9–65.3)	64.0 (59.1–68.6)	59.3 (54.5–64.0)	-0.9	0.740
Tennessee	66.7 (60.7–72.1)	73.6 (69.3–77.4)	67.6 (63.0–71.9)	0.9	0.745
Texas	65.5 (61.9–68.9)	63.8 (60.2–67.3)	61.7 (58.0–65.3)	-3.8	0.042
Utah	56.7 (53.8–59.5)	54.1 (51.6–56.5)	52.5 (49.9–55.1)	-4.2	0.039
Vermont	57.8 (53.5–62.0)	53.3 (49.2–57.4)	57.8 (53.4–62.2)	0.0	0.508
Virginia	67.5 (62.8–71.9)	65.7 (62.3–69.0)	62.9 (59.7–66.0)	-4.6	0.248
Washington	54.7 (51.4–57.9)	53.0 (50.1–55.9)	53.4 (50.6–56.1)	-1.3	0.219
West Virginia	73.8 (70.1–77.3)	68.1 (64.7–71.3)	67.2 (64.0–70.3)	-6.6	<0.001
Wisconsin	61.7 (55.7–67.3)	61.0 (55.8–66.0)	58.4 (53.4–63.2)	-3.3	0.070
Wyoming	57.3 (53.0–61.4)	57.8 (53.0–62.4)	56.4 (50.8–61.8)	-0.9	0.858

**Abbreviation:** CI = confidence interval.

* Directly standardized to the 2000 U.S. standard population.

^†^ Adjusted for sex, age group, and race/ethnicity.

^§^ Includes participants of multiple racial/ethnic groups.

Age-standardized estimates were lower than unadjusted estimates for self-reported hypertension (Supplementary Table 1; https://stacks.cdc.gov/view/cdc/50226) and substantially lower for antihypertension medication use (Supplementary Table 2; https://stacks.cdc.gov/view/cdc/50226). In addition, statistically significant increases were observed in the unadjusted prevalence of both hypertension (0.6%), and antihypertension medication use from 2011 to 2015; however, the increase in medication use was small in magnitude (0.1%).

## Discussion

Among U.S. adults, the age-standardized prevalence of self-reported hypertension and antihypertension medication use changed little from 2011 to 2015. Differences were observed by age, sex, race/ethnicity, and state of residence.

A recent report using National Health and Nutrition Examination Survey data found no change in the prevalence of hypertension among U.S. adults, from 1999–2000 (28.4%) to 2011–2012 (28.7%) and 2015–2016 (29.0%) (*4*). Because of the large number of participants in BRFSS each year, the statistically significant decline in hypertension prevalence from 30.1% to 29.8% likely does not represent a meaningful change. However, at the state level, both the age-standardized and unadjusted prevalences of hypertension declined significantly in Alaska, Michigan, Nevada, New Hampshire, and Texas and increased in Arkansas, Georgia, Hawaii, and West Virginia, which suggests that there might be notable changes in hypertension prevalence in these states.

The finding that the age-standardized prevalence of antihypertensive medication use declined slightly from 2011 (63.1%) to 2015 (61.8%) was unexpected, although the trend in unadjusted prevalence had no meaningful change (from 77.5% to 77.6%). A previous study found that hypertension medication prescriptions provided during U.S. physician office visits increased from 69.2% to 78.8% from 2003–2004 to 2009–2010 (*5*). U.S. prescription sales data also indicated that prescription fill counts for antihypertensive medication increased from 2009 to 2014 (*6*). Data from the National Health and Nutrition Examination Survey indicated that antihypertensive medication use increased from 63.5% (2001–2002) to 77.3% (2009–2010) (*7*).

Reduction targets in the prevalence of hypertension and improvements in its management are included in many national initiatives. *Healthy People 2020* heart disease and stroke objectives include reducing the proportion of persons in the population with hypertension (target = 26.9%) and increasing the proportion of adults with hypertension who are taking the prescribed medications to lower their blood pressure (target = 69.5%).^§^ Although improvements have been seen in hypertension management, *Healthy People 2020* hypertension targets have yet to be realized. Whereas *Healthy People 2020* objectives and targets are set for the United States, data from this report highlighting sociodemographic and geographic differences in the prevalence and treatment of hypertension can be used by state partners to target interventions to improve hypertension management within their populations and communities. Complementary to *Healthy People 2020* and other programs, the U.S. Department of Health and Human Services Million Hearts initiative^¶^ seeks to improve hypertension control through diverse, multifaceted interventions (*8*). CDC has been working with state and local public health communities to improve hypertension awareness, treatment, and control through multiple strategies within the CDC State Heart Disease and Stroke Prevention programs (*9*). In addition to effective, replicable interventions available through these programs, data from this report could be used by public health practitioners to inform hypertension awareness initiatives and management strategies with clinical partners.

The findings in this report are subject to at least three limitations. First, BRFSS data are based on self-report; the lack of direct blood pressure measurement makes it impossible to fully assess hypertension prevalence or control according to current guidelines. Based on data from the National Health and Nutrition Examination Survey, the prevalence of awareness among adults with hypertension was 83.3% during 2011–2014 (*10*). Therefore, nearly 20% of adults with hypertension are unaware of their condition. Second, the representativeness of the BRFSS sample might be affected by median response rates of <50% across the states. Finally, because hypertension is related to age, the slight decline in the age-standardized prevalence of medication use during the analysis period could be caused by the mathematical distortion of standardizing to a general population age distribution, or could reflect reporting bias.

This report provides the most current self-reported state-level hypertension surveillance data. Hypertension remains a significant public health problem. Public health and health system interventions might help to improve hypertension awareness and management. A substantial evidence base is available to inform programs at multiple levels and across diverse settings to support improvements in hypertension management.**^,††^

**Summary**

**What is already known about this topic?**

Hypertension is a major risk factor for heart disease and stroke. Hypertension prevalence and treatment among the U.S. population varies by demographic characteristics and by state.

**What is added by this report?**

During 2011–2015, overall, the age-standardized prevalence of hypertension (30.1% in 2011 to 29.8% in 2015), as well as the use of antihypertensive medication among persons with self-reported hypertension (63.0% in 2011 to 61.8% in 2015), decreased slightly among U.S. adults. However, it is unclear whether these small changes are clinically meaningful.

**What are the implications for public health practice?**

Aggressive public health actions to expand existing, effective interventions could enhance improvement in hypertension prevention and management in order to achieve *Healthy People 2020* goals.

## Footnotes

* https://www.cdc.gov/brfss.

† According to the guidelines for the American Association of Public Opinion Research.

§ https://www.healthypeople.gov/2020/topics-objectives/topic/heart-disease-and-stroke.

¶ https://millionhearts.hhs.gov/partners-progress/champions/index.html.

** https://www.thecommunityguide.org/topic/cardiovascular-disease.
